# Quercetin can be a more reliable treatment for metastatic prostate cancer than the localized disease: An in vitro study

**DOI:** 10.1111/jcmm.17783

**Published:** 2023-05-26

**Authors:** Akram Mirzaei, Roham Deyhimfar, Helia Azodian Ghajar, Rahil Mashhadi, Maryam Noori, Hossein Dialameh, Ziba Aghsaeifard, Seyed Mohammad Kazem Aghamir

**Affiliations:** ^1^ Urology Research Center Tehran University of Medical Sciences Tehran Iran; ^2^ Student Research Committee, School of Medicine Iran University of Medical Sciences Tehran Iran

**Keywords:** drug resistance, Osteopontin, prostate cancer, quercetin, vascular endothelial growth factor

## Abstract

Quercetin is a plant flavonoid that has been recognized to have anti‐inflammatory, antioxidant and anti‐proliferative activities. This study aims to evaluate the inhibitory effects of quercetin against prostate malignancy in vitro and the underlying resistance mechanism. IC_50_ values of quercetin were determined by MTT assay. Annexin‐V/PI staining was used to measure the rate of apoptosis. DNA cell cycle was analysed by PI staining method. Real‐time PCR was performed to assess mRNA levels of *OPN* isoforms, *VEGF* isoforms, *P53* and *KLK2*. Migration potential, proliferative capability and nucleus morphology of cells were evaluated by the scratch‐wound assay, colony‐forming assay and Hoechst staining, respectively. Quercetin significantly increased the apoptosis rate of PC‐3 and LNCaP cell lines, arrested the cell cycle at the sub‐G1/G1 phase, and reduced the migration potential and colony‐forming capability. Moreover, upregulation of apoptosis‐related genes and downregulation of genes involved in proliferation and angiogenesis was also observed. Although our results elucidated that quercetin has antitumor effects on PC‐3 and LNCaP, for the first time, we showed that quercetin treatment causes alterations in the expression of *OPN* and *VEGF* isoforms, which are cancer‐promoting modulators through various processes such as angiogenesis and drug‐resistance. Prostate malignant cells can dodge the anti‐carcinogenic properties of quercetin via modulation of *OPN* and *VEGF* isoforms in vitro. Therefore, quercetin acts as a double‐edged sword in prostate cancer treatment.

## 1 INTRODUCTION

According to Global Cancer Statistics in 2020, prostate cancer is the second most prevalent malignancy, responsible for 6.7% of cancer–related deaths in men all around the globe.^1^ The standard of care for prostate cancer at the early stages is radical prostatectomy and local radiotherapy, which mainly results in tumour recurrence and metastasis to body regions such as bones and lungs. Following metastasis, the patients are treated with systemic chemotherapy and androgen deprivation therapy (ADT).^2^ Despite the initial successful response, tumour cells become resistant to these treatments. Therefore, the disease relapses with a more aggressive phenotype, eventually leading to death.^3^ Considering these challenges, optimizing classic treatment approaches and establishing new strategies are highly demanding.

Plants' secondary metabolites are considered great candidates to improve standard cancer treatments. Quercetin (3, 3′, 4′, 5–7 pentahydroxyflavone) is a bioactive phenolic compound that is abundantly found in fruits and vegetables such as apples, onions and tea.^4^ Previous studies have shown that this compound has antioxidant,^5^ anti‐inflammatory^6^ and anti‐cancer properties.^7^ This antitumor activity can be exerted via several mechanisms such as alteration of tumour metabolism,^8^ inhibition of angiogenesis,^9^ improvement of tumours' chemosensitivity,^10^ inhibition of the epithelial‐to‐mesenchymal transition,^11^ decreasing tumour invasion potential through downregulation of matrix metalloproteinases,^12^ induction of apoptosis and inhibition of anti‐apoptotic pathways.^13^, ^14^ Despite its efficacy and minimal side effects, the clinical application of quercetin is limited due to its low bioavailability.^15^ Many successful pieces of research have been performed, focusing on formulating quercetin using innovative nano‐carriers to increase the bioavailability and therapeutic efficacy.^16^, ^17^ However, it is necessary to shed light on underlying mechanisms used by tumours to evade quercetin‐mediated anticancer effects to overcome post‐treatment resistance and progression.

Osteopontin (OPN) is a secretory glycoprotein from the Small Integrin‐Binding Ligand N‐linked Glycoprotein (SIBLING) family mainly produced by osteoblasts and osteoclasts to mediate the process of biomineralization.^18^ It has been recognized that OPN also has oncogenic roles either as a prognostic biomarker or a significant regulator of tumour progression.^19^, ^20^, ^21^, ^22^ OPN has three splicing isoforms: OPN‐a (full‐length protein with seven exons), OPN‐b and OPN‐c (lack Exons 5 and 4, respectively).^23^ Former studies have reported the overexpression of OPN‐b and OPN‐c in various cancer cell lines, mediating tumour survival, resistance to chemotherapeutic agents and, promoting angiogenesis.^24^, ^25^, ^26^, ^27^ However, the relationship between OPN and VEGF isoforms and quercetin in prostate cancer remains unclear. Therefore, our research aims to evaluate the antitumor potential of quercetin on LNCaP (Androgen‐sensitive, metastatic to lymph node) and PC‐3 cells (Androgen‐insensitive, metastatic to bone), which represents different stages of prostate cancer. LNCaP represents localized disease, while PC‐3 represents advanced disease. Quercetin‐mediated modulatory effects on OPN and VEGF will be evaluated as a potential mechanism for tumour resistance and progression.

## 2 MATERIALS AND METHODS

### 2.1 Cell culture

Cell lines of PC‐3 (ATCC Number: CRL‐1435) and LNCaP (ATCC Number: CRL‐10995) were obtained from the Pasteur Institute of Iran. Both cell lines maintained in DMEM medium (Gibco) supplemented with 10% fetal bovine serum (Gibco), 1000 units/mL Penicillin and 100 μg/mL streptomycin (Gibco) in a 5% CO_2_ humidified incubator at 37°C.

### 2.2 Cell proliferation assay

IC_50_ and inhibitory effects of quercetin on the metabolic activity of prostate cancer cell lines were evaluated using 3‐[4,5‐dimethylthiazol‐2‐yl]‐2,5 diphenyl tetrazolium bromide (MTT) assay. After seeding at 5 × 10^3^ cells/mL per well in 96‐well plates, cell lines were exposed to various concentrations of quercetin for 24 h, 48 h, and 72 h. Then, cells were incubated with 100 μL of MTT solution (0.5 mg/mL, Sigma‐Aldrich) for 4 h at 37°C. After the dissolving of formazan crystals in 100 μL of DMSO, the optical density was measured at the wavelength of 570 nm by an ELISA microplate reader. Dose–response curves were plotted and IC_50_ was graphed using Graph‐Pad PRISM software (v9).

Cell viability calculation formula (%) = [(average absorbance of triplicate treatment wells)/(average absorbance of control wells)] × 100.

### 2.3 3D cell colony formation assay

Cell lines in the treatment group and control group were seeded at the rate of 1.5 × 10^3^ cells per well on six‐well plates and incubated for 2 weeks. After 14 days, the bottom of the plate was coated by 2% agarose gel. Then, cells were mixed with the culture medium containing 0.7% agarose gel and poured on the gel‐coated surface of the plate. The culture medium was changed every 4 days. After this time, cells were fixed with cold formaldehyde, washed with PBS, and stained with 0.1% crystal violet. Calculation of the colony formation rate was done using ImageJ software.

### 2.4 Flow cytometric measurement of apoptosis

Cell viability, apoptosis and necrosis were determined using an Annexin‐V and PI (propidium iodide) kit according to the manufacturer's instructions.^28^ After overnight incubation of cell lines in DMEM/10% FBS at 37°C, cells were exposed to various concentrations of quercetin for 2 days. Incubation in darkness for 15 min at 37°C was done after the addition of PI and Annexin‐V, and then cells were analysed by flow cytometry device.

### 2.5 Staining of treated and control cells with Hoechst dye (33342)

Apoptosis was further assessed by the Hoechst staining method. Both cell lines were implanted in 24‐well plates (3 × 10^5^ cells per well), and incubated with various concentrations of quercetin for 48 h. Cells were treated with cold methanol (50 μL) for 20 min. After centrifugation, pellets were incubated with 100 μL PBS and 4 μL Hoechst dye for 20 min at 25°C in darkness, then observed under a fluorescence microscope (100× magnification).

### 2.6 Migration potential analysis by Scratch‐wound assay

A vertical scratch was applied via pipette tip to the confluent PC‐3 and LNCaP cells (about 85% confluency) and the plates were washed with serum‐free medium twice. After overnight serum starvation, control and experimental groups were exposed to PBS and quercetin, respectively. Finally, cell imaging was performed at 24 h intervals. The cell migration rate was estimated by measurement of area among the two scratch edges in comparison to the control group.

### 2.7 DNA cell cycle analysis

Quercetin‐treated (48 h) and untreated cells were fixed using 70% cold ethanol for 24 h. After double wash with PBS, cells were incubated with RNase I and 500 μL PI for 30 min at 37°C. Cell detection was performed by flow cytometer. Flowjo software was used to analyse the data. Cell arrest at sub‐G0/G1 was considered apoptosis.

### 2.8 Gene expression analysis by real‐time PCR

Total RNA extraction was performed using TriPure Isolation Reagent. Colibri Microvolume Spectrometer was used to determine the RNA concentration. cDNA was generated using Takara cDNA synthesis kit. Real‐time PCR was performed using QIAGEN's thermocycler with a total sample volume of 20 μL. The PCR reaction specificity confirmation was applied through melting curve analysis. GAPDH mRNA levels were considered as an internal control to estimate the relative expression levels by the 2^−ΔΔCT^ method.^29^ Table 1 represents the nucleotide sequences of primers.

**TABLE 1:** Nucleotide sequence of specific primers for use in real‐time PCR technique.

Gene	Accession number	Forward primer (5′–3′)	Reverse primer (5′–3′)
*OPN‐a*	NM_001040058.1	ATCTCCTAGCCCCACAGCAAT	CATCAGACTGGTGAGAATCATC
*OPN‐b*	NM_000582.2	ATCTCCTAGCCCCAGACGAC	AAAATCAGTGACCAGTTCATCAG
*OPN‐c*	NM_001040060.1	TGAGGAAAAGCAGAATGCCTG	GTCAATGGAGTCCTGGCTGT
*VEGF‐a*	NM_001316955.1	CTCACCAAGGCCAGCACATAGG	ATCTGGTTCCGAAAACCCTGAG
*VEGF‐c*	NM_005429.4	GTCTGTGTCCAGTGTAGATG	AGGTAGCTCGTGCTGGTGTT
*KLK2*	NM_005551	TCAAGGGTGAGCCCTTTCACT	ATCCTCTCCCTTTCCCTCAT
*TP53*	NM‐011640	AGACCTATGGAAACTACCTTC	GGACAGCATCAAATCATC
*GAPDH*	NM‐001289746.1	GTGAACCATGAGAAGTATGACCAAC	CATGAGTCCTTCCACGATACC

### 2.9 Statistical analysis

All experiments were performed in triplicate and the data were presented as means ± SD. Statistical analysis was performed by anova and Student's *t*‐test. Statistical significance was considered as *p < 0.05, **p < 0.01, ***p < 0.001, and ****p < 0.0001.

## 3 RESULTS

### 3.1 Morphological changes

**FIGURE 1:**
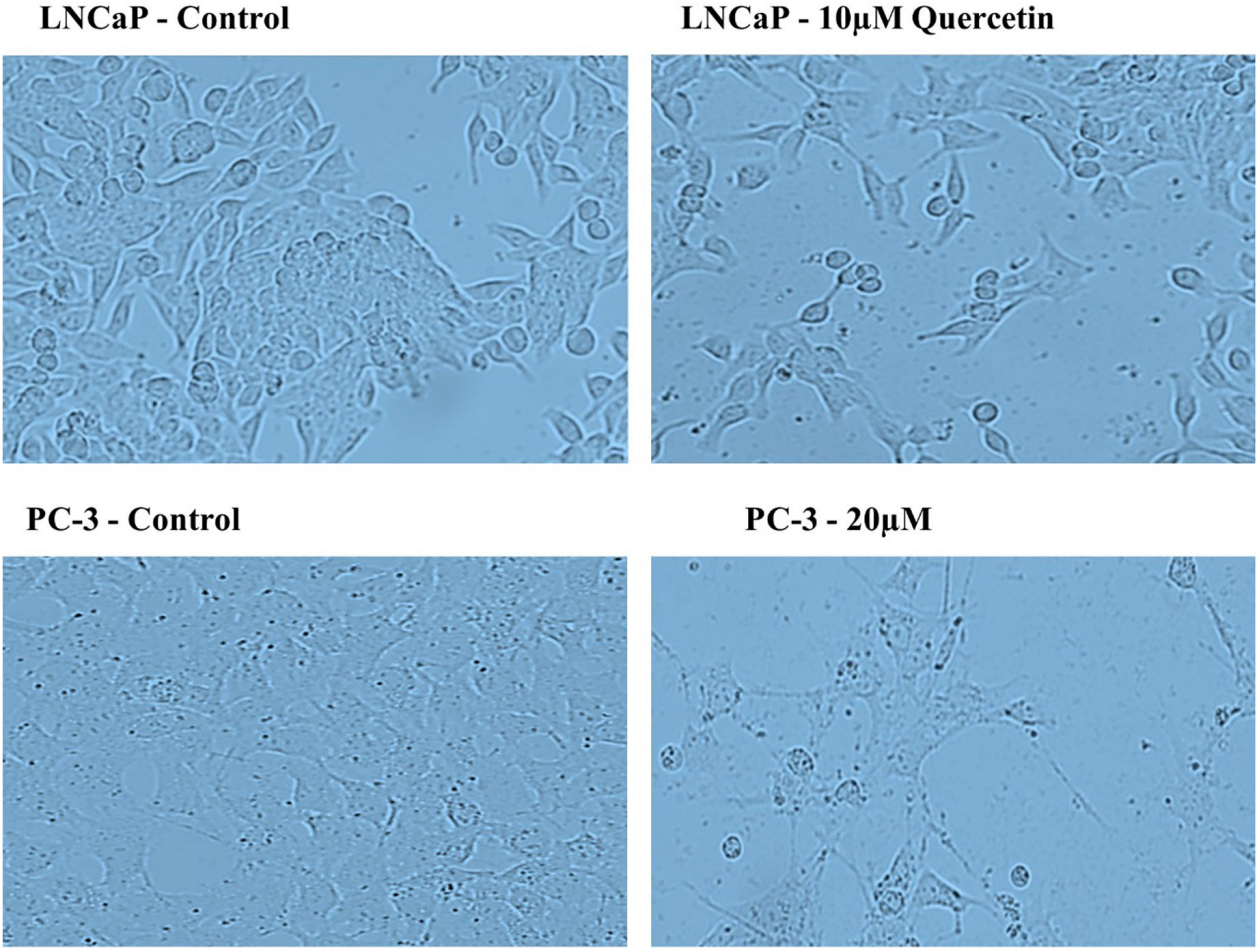
Light microscopic images of PC‐3 and LNCaP cells in the control group and treated group with quercetin.

**FIGURE 2:**
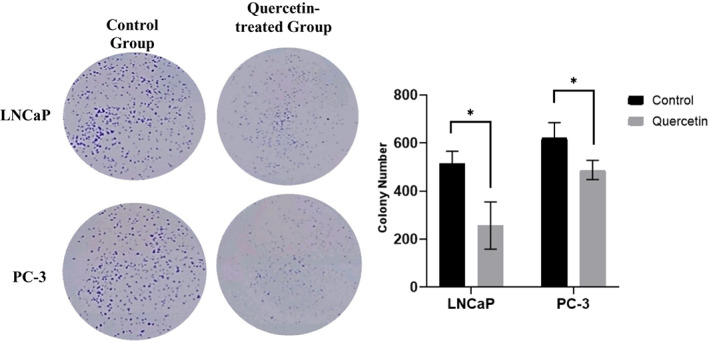
3D Colony formation assay in LNCaP and PC‐3 prostate cancer cells. Quercetin treatment of LNCaP (10 μM) and PC‐3 cells (20 μM) impelled a significant decrease in the colony number.

Quercetin‐related alterations in the morphology of prostate cancer cell lines were observed through an inverted microscope (Figure 1). Cells underwent modifications such as membrane protrusion, shrinkage and rounding, indicating that quercetin can prompt apoptosis in malignant prostate cells. Also, a significant decrease in the number of colonies commends that quercetin can effectively inhibit cellular proliferation (Figure 2).

#### 3.1.1 Quercetin prevents cell viability of PC‐3 and LNCaP cell lines in a time‐ and dose‐dependent manner

**FIGURE 3:**
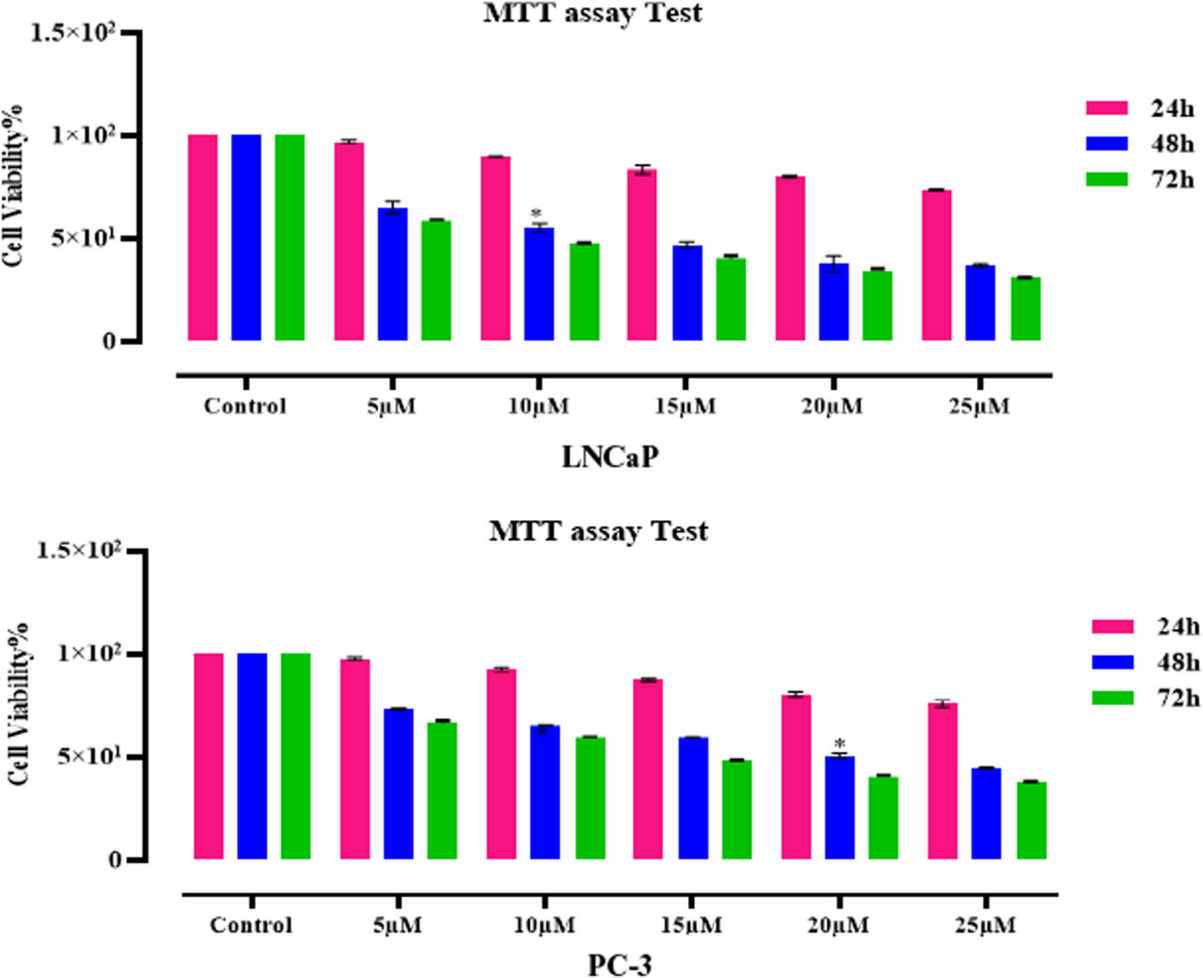
In both cell lines, the cytotoxic effects of quercetin (0‐25 μM) were studied and the anti‐proliferative properties of these quercetin concentrations were evaluated by MTT assay. IC_50_ values for quercetin were determined as 20 μM and 10 μM for PC‐3 and LNCaP, respectively. Quercetin significantly exerted cytotoxicity on cancer cells in dose‐and time‐dependent manners.

In both cell lines, the cytotoxic effects of quercetin (0–25 μM) were studied and the anti‐proliferative properties of this phytochemical were evaluated by MTT assay (Figure 3). IC_50_ values were determined as 20 μM and 10 μM for PC‐3 and LNCaP, respectively. Quercetin significantly exerted cytotoxicity on cancer cells in dose‐and time‐dependent manners.

#### 3.1.2 Effects of quercetin on the programmed cell death (apoptosis)

**FIGURE 4:**
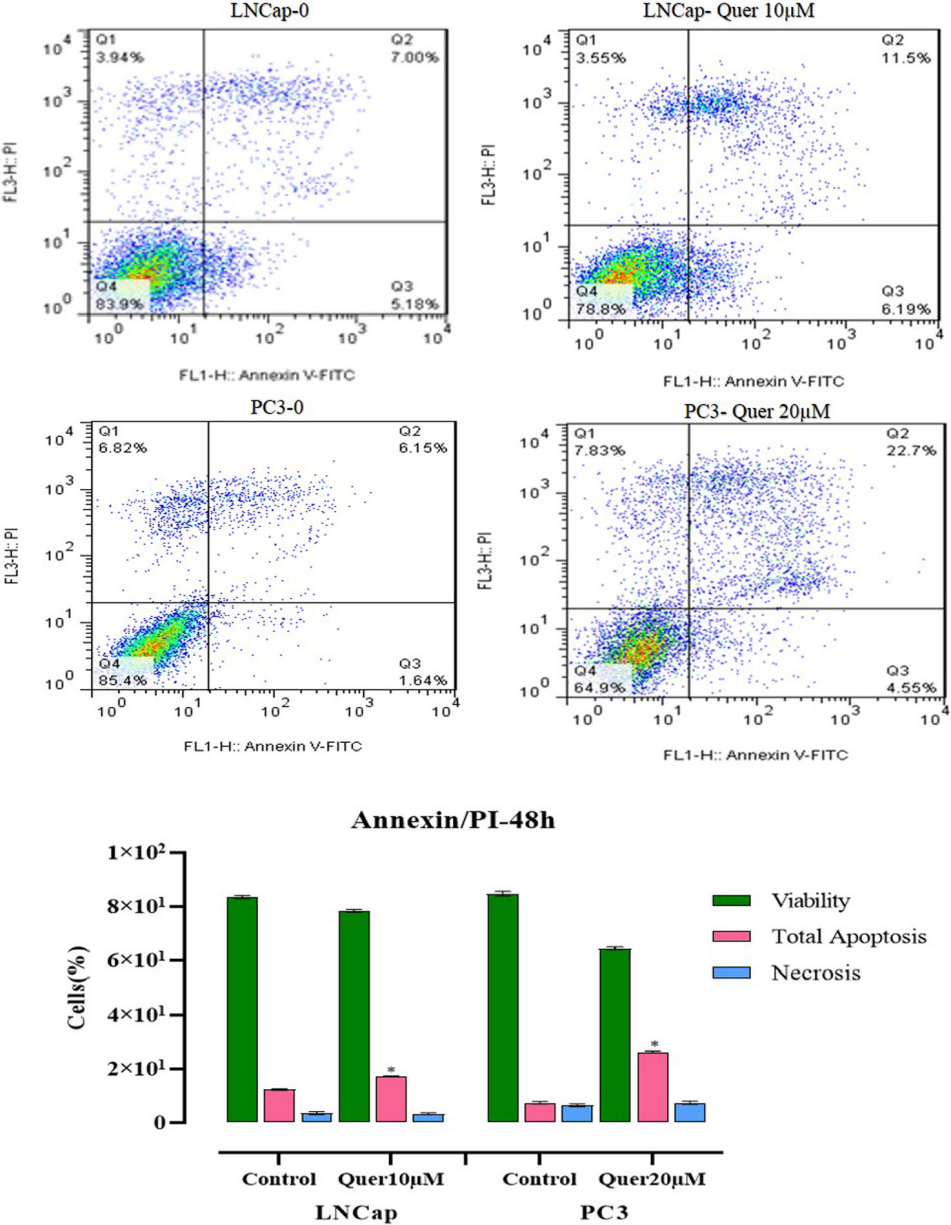
Analysis of viability, apoptosis and necrosis with flow cytometry. Lower left, lower right, upper right and upper left quadrants show live cells (PI−/FITC−), early apoptosis (PI−/FITC+), late apoptosis (PI+/FITC+), and necrosis (PI+/FITC−), respectively.

Annexin‐V/PI staining was executed to determine whether quercetin can prompt apoptosis of prostate cancer cell lines. Quercetin exposure for 48 h augmented the rate of apoptotic cell death (*p* < 0.01), indicating that quercetin could ameliorate apoptosis in prostate carcinoma in vitro (Figure 4).

#### 3.1.3 Quercetin arrested prostate cancer cells at SubG1/G1 phase

**FIGURE 5:**
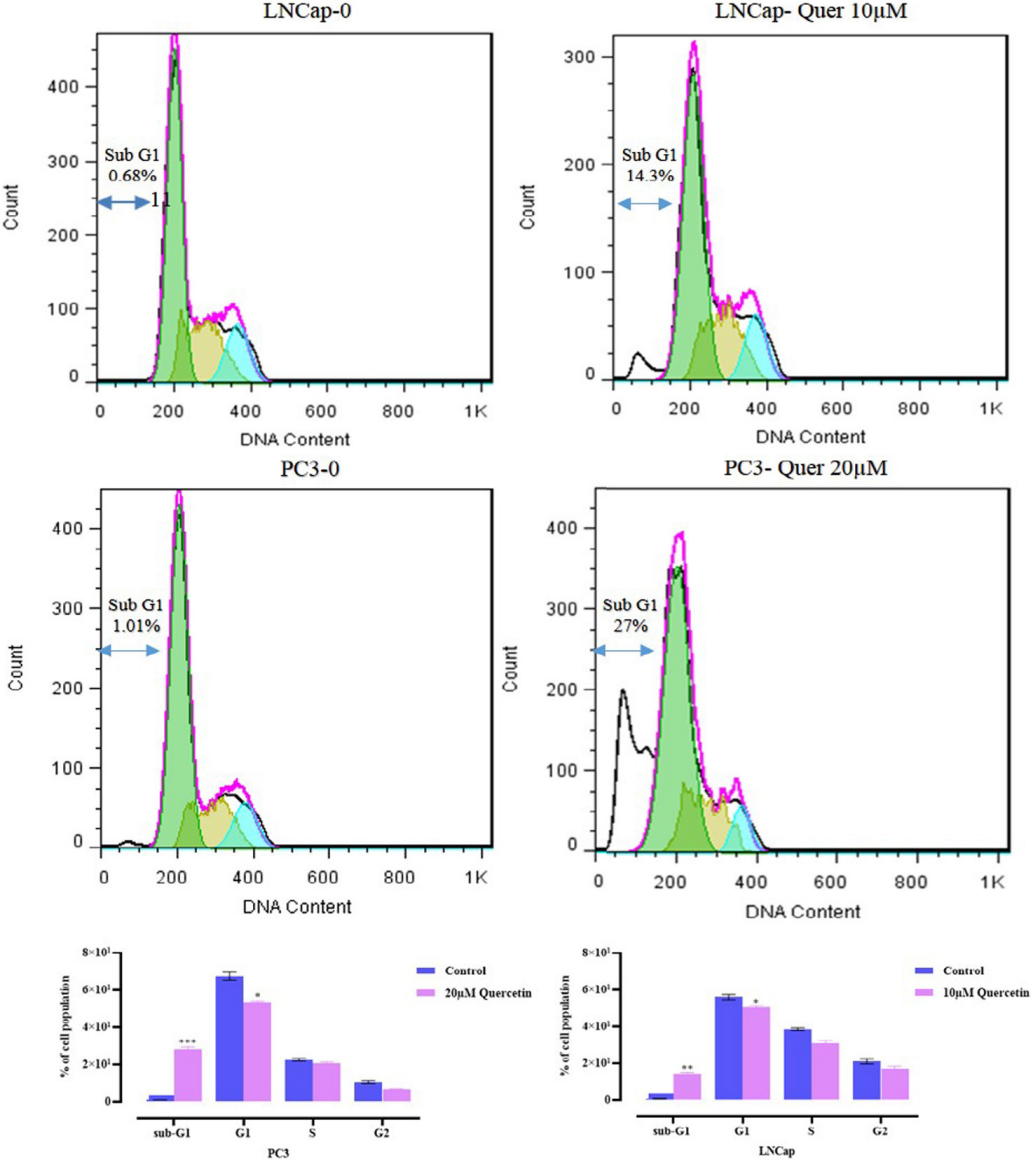
Analysis of cell cycle for LNCaP and PC‐3 cell lines.

The impact of quercetin on cell cycle arrest of prostate cancer cell lines was assessed by flow cytometry analysis (Figure 4). Our data revealed a significant increase in proportion of both cell lines arrested at the SubG1/G1 phase in a dose‐dependent manner. Post‐treatment accumulation of cells at sub‐G1phase can be considered as a demonstration of apoptosis. Therefore, our results indicated that quercetin‐related arrest of the cell cycle eventually led to apoptosis compared to the control group (Figure 5).

#### 3.1.4 Effects of quercetin on nucleus and cellular migration

**FIGURE 6:**
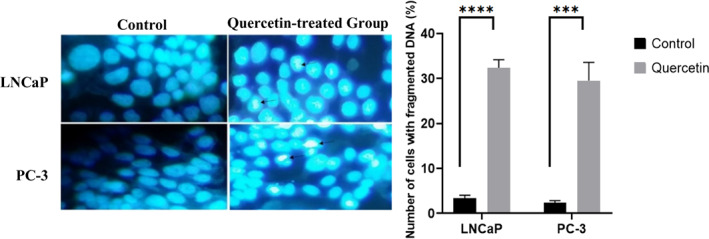
Fluorescent staining with Hoechst dye (33342). Both cell lines were treated with 10 μM and 20 μM quercetin, respectively. Fragmented nuclei in some cells indicate apoptosis.

**FIGURE 7:**
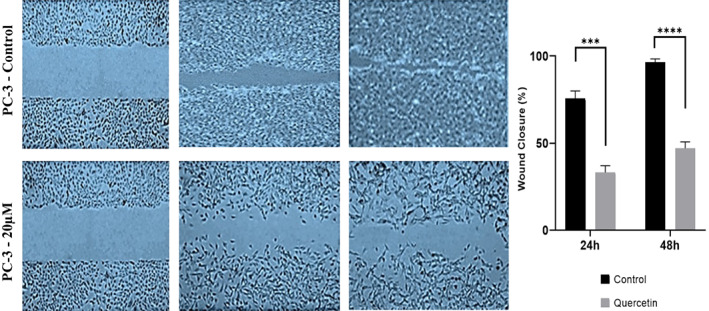
Migration assay for PC‐3 prostate cancer cells after exposing to 20 μM of quercetin. The cells' movement into the wounds was revealed at 0, 24 and 48 h under the 100× magnification.

Fluorescent staining with Hoechst 33342 demonstrated significant variations in the nuclei morphology upon quercetin treatment (Figure 6). Dispersed nuclear contents observed under the fluorescent microscope represent apoptosis. Additionally, quercetin has intensely interdicted PC‐3 cell migration after quercetin treatment (20 μM) (Figure 7).

#### 3.1.5 Quercetin treatment alters gene expression profile of LNCaP and PC‐3 cells

**FIGURE 8:**
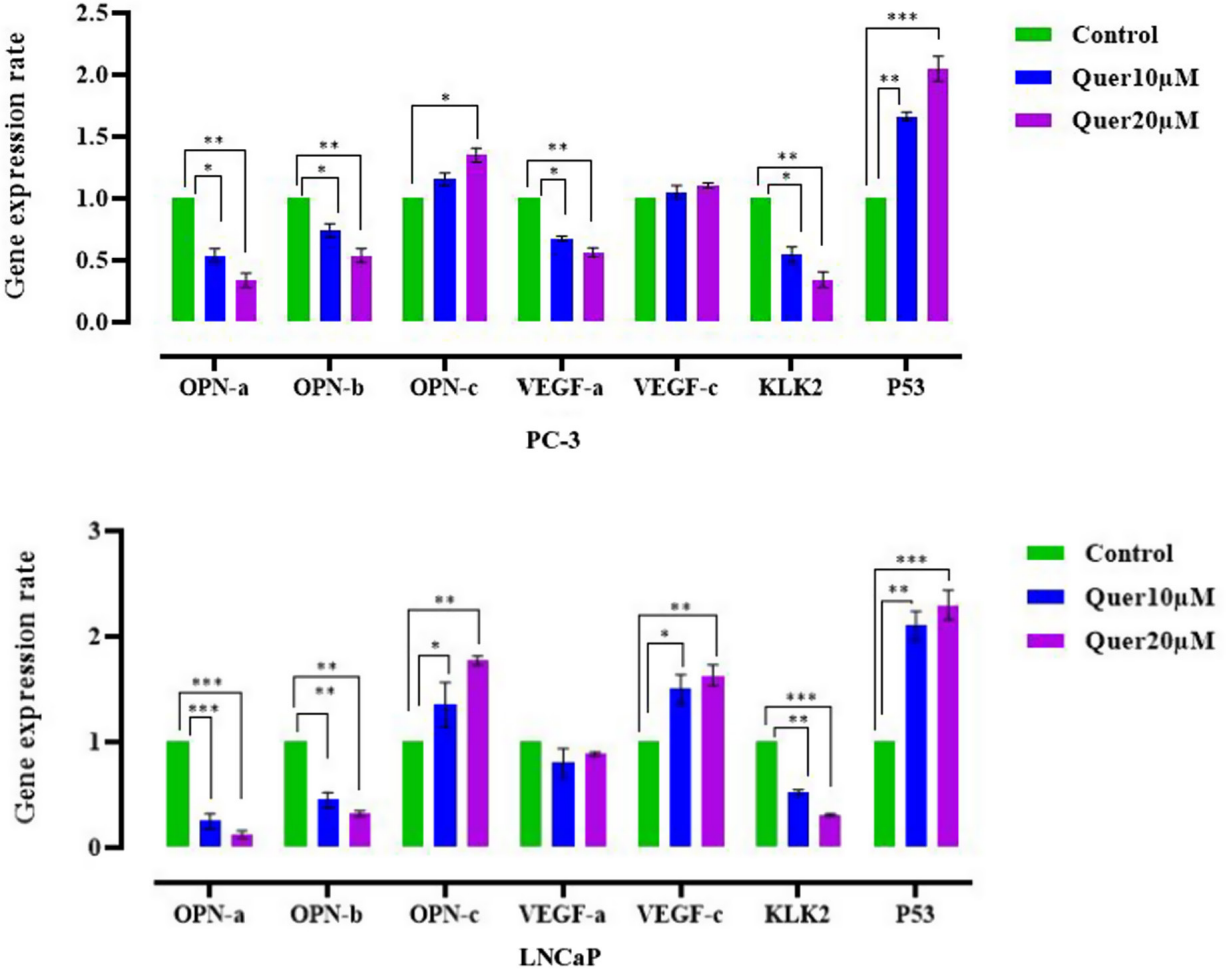
Assessment of changes in mRNA level of *OPN‐a*, *OPN‐b*, *OPN‐c* and *VEGF*, *KLK2* and *P53* genes in quercetin‐treated (48 h) prostate cancer cell lines by qRT‐PCR.

Both quercetin‐treated cell lines (48 h) were studied using real‐time PCR for evaluation of *VEGF* isoforms (a and c) as angiogenesis‐related markers, *OPN* isoforms (a, b and c) as a clinically useful biomarker of tumour progression, *P53* for apoptosis and Kallikrein Related Peptidase 2 (*KLK2*) as a prognostic marker for prostate cancer risk. Levels of *P53* expression significantly increased in all experimental groups compared to the control (*p* < 0.001). Gene expression levels of *OPN‐a* and *OPN‐b*, and ad *KLK2* downregulated sharply and the expression of OPN‐c upregulated in all experimental groups compared to the control (*p* < 0.01). While the level of the *VEGF‐c* isoform increased in quercetin‐treated LNCaP cells (*p* < 0.01), level of *VEGF‐a* isoform decreased in treated PC‐3 cells (*p* < 0.01). Therefore, prostate cancer cells escape treatment by modulating the expression of mentioned genes (Figure 8).

## 4 DISCUSSION

Despite recent advances in the diagnosis and treatment, prostate cancer has remained a significant challenge in terms of prevalence and mortality.^1^ Various treatments for prostate cancer still suffer from two major problems: side effects and tumour resistance. Naturally‐occurring compounds are perfect candidates to overcome these problems and improve treatment outcomes.^30^ To date, a large body of research has demonstrated the beneficial effects of quercetin as a potent phytochemical to fight prostate cancer. A study by Xing et al. revealed that treating LNCaP cells (lymph node metastases and androgen‐sensitive) causes downregulation of the androgen receptor gene in these cells and weakens its related functions.^31^ Furthermore, quercetin as a chemo‐sensitizer agent has shown promising results in prostate cancer. In a study by Tummala et al., quercetin broke the resistance of human prostate cancer cells to the drug enzalutamide by targeting hnRNPA1.^32^ In another study by Wang et al., combined treatment with quercetin and docetaxel arrested the cell cycle at the G2/M phase and induced significant apoptosis.^33^ In another study by Lu et al., the exposure of docetaxel‐sensitive prostate cancer cells to docetaxel/quercetin led to inhibition of the PI3K/Akt signalling pathway and AR‐mediated apoptosis.^34^ Matrix metalloproteinases (MMP) 2 and 9 are among the main factors facilitating prostate cancer metastasis. A study conducted by Vijayababu et al. showed that MMP‐2 and MMP‐9 were downregulated in PC‐3 cells after treatment with multiple doses of quercetin. Subsequently, the invasion potential of prostate cancer was also lessened.^12^ Sun et al., also demonstrated that the combined use of metformin and quercetin synergistically suppressed the prostate cancer cells through the VEGF/PI3K/Akt axis.^35^

According to the body of evidence from previous studies, human *kallikrein‐related peptidase 2 (KLK2)*, a protease that is exclusively expressed in the prostate gland, can facilitate prostate cancer progression and act as a biomarker.^36^, ^37^ It has been reported that during prostate cancer, upregulation of the *KLK2* gene is associated with cell proliferation, migration, invasion, angiogenesis and apoptosis resistance of malignant cells.^38^, ^39^ Therefore, *KLK2* has been used in many studies as a biological prostate cancer prognosis marker as well as a therapeutic target. Quercetin exposure resulted in a dramatic decrease of *KLK2* mRNA expression in both prostate cancer cell lines, endorsing the tumour‐inhibiting characteristics of this compound.

The role of *OPN* isoforms in tumour resistance and cancer progression is heavily under investigation. It was found by Tilli et al. that *OPN‐b* and *OPN‐c* isoforms are upregulated in prostate cancers with tumorigenic effects such as inducing cell proliferation, tumour in vivo growth, migration, invasion and suppressing colony formation.^24^ It has been shown that *OPN* expression has a strong association with the expression of hypoxia‐inducible factor 1 alpha (HIF‐1a) and VEGF via Akt/ILK/NF‐kB/ ATF‐4/PI3K/αvβ3 integrin/ERK1/2 pathway.^40^, ^41^

According to previous studies, the role of *VEGF* isoforms in tumour microenvironments might be beyond angiogenesis and lymphangiogenesis.^29^ Maxwell et al. illustrated that co‐inhibition of *VEGF‐a* and IL‐8 sensitizes enzalutamide‐resistant prostate cancer cells to this drug.^42^ Other research conducted by Yang and Jenbacken has demonstrated that the *VEGF‐c* expression is associated with prostate cancer progression and metastasis to lymph nodes.^42^, ^43^ Our results indicate that the androgen signalling pathway may play a role in quercetin‐mediated alteration in the expression of *VEGF* isoforms. Quercetin treatment in androgen‐dependent LNCaP cells resulted in the upregulation of *VEGF‐c*, without any significant impact on *VEGF‐a* expression. Therefore, it can be concluded that, despite quercetin's favourable anti‐cancer properties, such treatment may exacerbate prostate cancer progression via upregulated *VEGF‐c* isoform in remaining tumour cells. Interestingly, in androgen‐independent PC‐3 cells, quercetin treatment significantly decreased in the *VEGF‐a* but not *VEGF‐c*. Considering the role of *VEGF‐a* in prostate cancer progression and drug resistance, patients with advanced disease may benefit from quercetin. These results indicate that quercetin might be useful for prostate cancer treatment exclusively in advanced conditions. In the early stages of the disease, quercetin may assist treatment‐survived cancer cells, leading to an enhanced malignant phenotype. More comprehensive studies are necessary to confirm and validate our findings.

There are detailed subjects that we aim to consider in our forthcoming research. For example, we would investigate the effects of quercetin specifically on cancer stem cells since this tumour sub‐population plays a vital role in disease relapse with a resistant phenotype. Moreover, our data can be deeply validated by knockdown/overexpress experiments using RNAi and viral‐based gene therapies. To obtain more reliable data, the brain‐metastatic DU145 cell line can also be investigated along with PC‐3 and LNCaP. In addition, future studies can include xenograft murine models for evaluation of quercetin‐resistance of prostate cancer cells in vivo. Western blot technique can be used to analyse the expression of *OPN* and *VEGF* isoforms at the protein level.

## 5 CONCLUSION

In the current study, we reconfirmed that quercetin has anti‐tumour properties on PC‐3 and LNCaP cell lines by decreasing cell survival, attenuating migration potential, induction of apoptosis and cell cycle arrest. The gene expression of *OPN* isoforms, *VEGF* isoforms, *P53* and *KLK2* was also modified following the quercetin treatment. In both cell lines, *OPN‐A, OPN‐B* and *KLK2* were significantly downregulated, while *p53* was significantly upregulated. VEGF‐c exhibited a significant upregulation only in LNCaP cells, while significant downregulation of VEGF‐a was exclusively seen in PC‐3 cells. Based on our findings, we propose that quercetin treatment is an ideal option for advanced prostate cancer but not localized disease.

## AUTHOR CONTRIBUTIONS

**Akram Mirzaei:** Methodology (equal); writing – original draft (equal). **Roham Deyhimfar:** Writing – original draft (equal); writing – review and editing (equal). **Helia Azodian Ghajar:** Methodology (equal); resources (equal). **Rahil Mashhadi:** Data curation (equal). **Maryam Noori:** Formal analysis (equal). **Hossein Dialameh:** Visualization (equal). **Ziba Aghsaeifard:** Validation (equal). **Seyed Mohammad Kazem Aghamir:** Conceptualization (equal).

## CONFLICT OF INTEREST STATEMENT

The authors confirm that there are no conflicts of interest.

## Data Availability

Information, data, and photos will be provided if requested.

## References

[1] Sung H , Ferlay J , Siegel RL , et al. Global cancer statistics 2020: GLOBOCAN estimates of incidence and mortality worldwide for 36 cancers in 185 countries. CA Cancer J Clin. 2021;71(3):209‐249. 33538338 10.3322/caac.21660

[2] Teo MY , Rathkopf DE , Kantoff P . Treatment of advanced prostate cancer. Annu Rev Med. 2019;70:479‐499. 30691365 10.1146/annurev-med-051517-011947 PMC6441973

[3] Heidenreich A , Bastian PJ , Bellmunt J , et al. EAU guidelines on prostate cancer. Part II: treatment of advanced, relapsing, and castration‐resistant prostate cancer. Eur Urol. 2014;65(2):467‐479. 24321502 10.1016/j.eururo.2013.11.002

[4] Azeem M , Hanif M , Mahmood K , Ameer N , Chughtai FRS , Abid U . An insight into anticancer, antioxidant, antimicrobial, antidiabetic and anti‐inflammatory effects of quercetin: a review. Polym Bull (Berl). 2023;80(1):241‐262. 35125574 10.1007/s00289-022-04091-8 PMC8800825

[5] Remigante A , Spinelli S , Straface E , et al. Antioxidant activity of quercetin in a H2O2‐induced oxidative stress model in red blood cells: functional role of band 3 protein. Int J Mol Sci. 2022;23(19):10991. 36232293 10.3390/ijms231910991 PMC9569818

[6] Hsieh H‐L , Yu M‐C , Cheng L‐C , et al. Quercetin exerts anti‐inflammatory effects via inhibiting tumor necrosis factor‐α‐induced matrix metalloproteinase‐9 expression in normal human gastric epithelial cells. World J Gastroenterol. 2022;28(11):1139‐1158. 35431500 10.3748/wjg.v28.i11.1139 PMC8985486

[7] Caruana R , Montalbano F , Zizzo MG , et al. Enhanced anticancer effect of quercetin microparticles formulation obtained by spray drying. Int J Food Sci Technol. 2022;57(5):2739‐2746.

[8] Wang L , Ji S , Liu Z , Zhao J . Quercetin inhibits glioblastoma growth and prolongs survival rate through inhibiting glycolytic metabolism. Chemotherapy. 2022;67(3):132‐141. 35249013 10.1159/000523905

[9] Zhao X , Wang Q , Yang S , et al. Quercetin inhibits angiogenesis by targeting calcineurin in the xenograft model of human breast cancer. Eur J Pharmacol. 2016;781:60‐68. 27041643 10.1016/j.ejphar.2016.03.063

[10] Lee SH , Lee EJ , Min KH , et al. Quercetin enhances chemosensitivity to gemcitabine in lung cancer cells by inhibiting heat shock protein 70 expression. Clin Lung Cancer. 2015;16(6):e235‐e243. 26050647 10.1016/j.cllc.2015.05.006

[11] Elumalai P , Ezhilarasan D , Raghunandhakumar S . Quercetin inhibits the epithelial to mesenchymal transition through suppressing Akt mediated nuclear translocation of β‐catenin in lung cancer cell line. Nutr Cancer. 2022;74(5):1894‐1906. 34338101 10.1080/01635581.2021.1957487

[12] Vijayababu M , Arunkumar A , Kanagaraj P , Venkataraman P , Krishnamoorthy G , Arunakaran J . Quercetin downregulates matrix metalloproteinases 2 and 9 proteins expression in prostate cancer cells (PC‐3). Mol Cell Biochem. 2006;287:109‐116. 16645725 10.1007/s11010-005-9085-3

[13] Chien S‐Y , Wu Y‐C , Chung J‐G , et al. Quercetin‐induced apoptosis acts through mitochondrial‐and caspase‐3‐dependent pathways in human breast cancer MDA‐MB‐231 cells. Hum Exp Toxicol. 2009;28(8):493‐503. 19755441 10.1177/0960327109107002

[14] Ward AB , Mir H , Kapur N , Gales DN , Carriere PP , Singh S . Quercetin inhibits prostate cancer by attenuating cell survival and inhibiting anti‐apoptotic pathways. World J Surg Oncol. 2018;16(1):1‐12. 29898731 10.1186/s12957-018-1400-z PMC6001031

[15] Lin J , Teo LM , Leong LP , Zhou W . In vitro bioaccessibility and bioavailability of quercetin from the quercetin‐fortified bread products with reduced glycemic potential. Food Chem. 2019;15(286):629‐635. 10.1016/j.foodchem.2019.01.199 30827656

[16] Minaei A , Sabzichi M , Ramezani F , Hamishehkar H , Samadi N . Co‐delivery with nano‐quercetin enhances doxorubicin‐mediated cytotoxicity against MCF‐7 cells. Mol Biol Rep. 2016;43:99‐105. 26748999 10.1007/s11033-016-3942-x

[17] Yadav N , Tripathi AK , Parveen A . PLGA‐quercetin nano‐formulation inhibits cancer progression via mitochondrial dependent caspase‐3, 7 and independent FoxO1 activation with concomitant PI3K/AKT suppression. Pharmaceutics. 2022;14(7):1326. 35890222 10.3390/pharmaceutics14071326 PMC9323198

[18] Icer MA , Gezmen‐Karadag M . The multiple functions and mechanisms of osteopontin. Clin Biochem. 2018;59:17‐24. 30003880 10.1016/j.clinbiochem.2018.07.003

[19] Rud AK , Boye K , Øijordsbakken M , et al. Osteopontin is a prognostic biomarker in non‐small cell lung cancer. BMC Cancer. 2013;13:1‐10. 24215488 10.1186/1471-2407-13-540 PMC3830440

[20] Agrawal D , Chen T , Irby R , et al. Osteopontin identified as lead marker of colon cancer progression, using pooled sample expression profiling. J Natl Cancer Inst. 2002;94(7):513‐521. 11929952 10.1093/jnci/94.7.513

[21] Khatami F , Aghamir SMK , Salmaninejad A , Shivarani S , Khorrami MH . Biomarkers for prostate cancer diagnosis from genetic perspectives. Transl Res Urol. 2020;2(2):51‐58.

[22] Tamehri Zadeh SS , Taheri D , Shivarani S , Khatami F , Kazemi R . Liquid biopsy in prostate cancer diagnosis and prognosis: a narrative review. Transl Res Urol. 2020;2(4):139‐146.

[23] Gimba E , Tilli T . Human osteopontin splicing isoforms: known roles, potential clinical applications and activated signaling pathways. Cancer Lett. 2013;331(1):11‐17. 23246372 10.1016/j.canlet.2012.12.003

[24] Tilli TM , Mello KD , Ferreira LB , et al. Both osteopontin‐c and osteopontin‐b splicing isoforms exert pro‐tumorigenic roles in prostate cancer cells. Prostate. 2012;72(15):1688‐1699. 22495819 10.1002/pros.22523

[25] Ivanov SV , Ivanova AV , Goparaju CM , Chen Y , Beck A , Pass HI . Tumorigenic properties of alternative osteopontin isoforms in mesothelioma. Biochem Biophys Res Commun. 2009;382(3):514‐518. 19285954 10.1016/j.bbrc.2009.03.042

[26] Nakamura K , Tilli T , Wanderley J , et al. Osteopontin splice variants expression is involved on docetaxel resistance in PC3 prostate cancer cells. Tumour Biol. 2016;37:2655‐2663. 26404131 10.1007/s13277-015-4095-6

[27] Mirzaei A , Mohammadi S , Ghaffari SH , et al. Osteopontin b and c isoforms: molecular candidates associated with leukemic stem cell chemoresistance in acute myeloid leukemia. Asian Pac J Cancer Prev. 2017;18(6):1707‐1715. 28670893 10.22034/APJCP.2017.18.6.1707 PMC6373801

[28] Arur S , Uche UE , Rezaul K , et al. Annexin I is an endogenous ligand that mediates apoptotic cell engulfment. Dev Cell. 2003;4(4):587‐598. 12689596 10.1016/s1534-5807(03)00090-x

[29] Mirzaei A , Rashedi S , Akbari MR , Khatami F , Aghamir SMK . Combined anticancer effects of simvastatin and arsenic trioxide on prostate cancer cell lines via downregulation of the VEGF and OPN isoforms genes. J Cell Mol Med. 2022;26(9):2728‐2740. 35366048 10.1111/jcmm.17286 PMC9077302

[30] Choudhari AS , Mandave PC , Deshpande M , Ranjekar P , Prakash O . Phytochemicals in cancer treatment: from preclinical studies to clinical practice. Front Pharmacol. 2020;10:1614. 32116665 10.3389/fphar.2019.01614 PMC7025531

[31] Xing N , Chen Y , Mitchell SH , Young CY . Quercetin inhibits the expression and function of the androgen receptor in LNCaP prostate cancer cells. Carcinogenesis. 2001;22(3):409‐414. 11238180 10.1093/carcin/22.3.409

[32] Tummala R , Lou W , Gao AC , Nadiminty N . Quercetin targets hnRNPA1 to overcome enzalutamide resistance in prostate cancer CellsQuercetin targets hnRNPA1 and synergizes with enzalutamide. Mol Cancer Ther. 2017;16(12):2770‐2779. 28729398 10.1158/1535-7163.MCT-17-0030 PMC5716891

[33] Wang P , Henning SM , Heber D , Vadgama JV . Sensitization to docetaxel in prostate cancer cells by green tea and quercetin. J Nutr Biochem. 2015;26(4):408‐415. 25655047 10.1016/j.jnutbio.2014.11.017 PMC4375039

[34] Lu X , Yang F , Chen D , et al. Quercetin reverses docetaxel resistance in prostate cancer via androgen receptor and PI3K/Akt signaling pathways. Int J Biol Sci. 2020;16(7):1121‐1134. 32174789 10.7150/ijbs.41686 PMC7053318

[35] Sun S , Gong F , Liu P , Miao Q . Metformin combined with quercetin synergistically repressed prostate cancer cells via inhibition of VEGF/PI3K/Akt signaling pathway. Gene. 2018;664:50‐57. 29678660 10.1016/j.gene.2018.04.045

[36] Shang Z , Niu Y , Cai Q , et al. Human kallikrein 2 (KLK2) promotes prostate cancer cell growth via function as a modulator to promote the ARA70‐enhanced androgen receptor transactivation. Tumour Biol. 2014;35:1881‐1890. 24122203 10.1007/s13277-013-1253-6

[37] Diamandis EP , Yousef GM , Luo L‐Y , Magklara A , Obiezu CV . The new human kallikrein gene family: implications in carcinogenesis. Trends Endocrinol Metab. 2000;11(2):54‐60. 10675891 10.1016/s1043-2760(99)00225-8

[38] Kyte JA . Strategies for improving the efficacy of CAR T cells in solid cancers. Cancers. 2022;14(3):571. 35158839 10.3390/cancers14030571 PMC8833730

[39] Bonk S , Kluth M , Jansen K , et al. Reduced KLK2 expression is a strong and independent predictor of poor prognosis in ERG‐negative prostate cancer. Prostate. 2020;80(13):1097‐1107. 32628300 10.1002/pros.24038

[40] Pang X , Gong K , Zhang X , Wu S , Cui Y , Qian B‐Z . Osteopontin as a multifaceted driver of bone metastasis and drug resistance. Pharmacol Res. 2019;144:235‐244. 31028902 10.1016/j.phrs.2019.04.030

[41] Qian J , LeSavage BL , Hubka KM , et al. Cancer‐associated mesothelial cells promote ovarian cancer chemoresistance through paracrine osteopontin signaling. J Clin Invest. 2021;131(16):e146186. 34396988 10.1172/JCI146186 PMC8363279

[42] Maxwell PJ , McKechnie M , Armstrong CW , et al. Attenuating adaptive VEGF‐A and IL8 signaling restores durable tumor control in AR antagonist–treated prostate cancers. Mol Cancer Res. 2022;20(6):841‐853. 35302608 10.1158/1541-7786.MCR-21-0780 PMC9381111

[43] Yang J , Wu HF , Qian LX , et al. Increased expressions of vascular endothelial growth factor (VEGF), VEGF‐C and VEGF receptor‐3 in prostate cancer tissue are associated with tumor progression. Asian J Androl. 2006;8(2):169‐175. 16491267 10.1111/j.1745-7262.2006.00120.x

