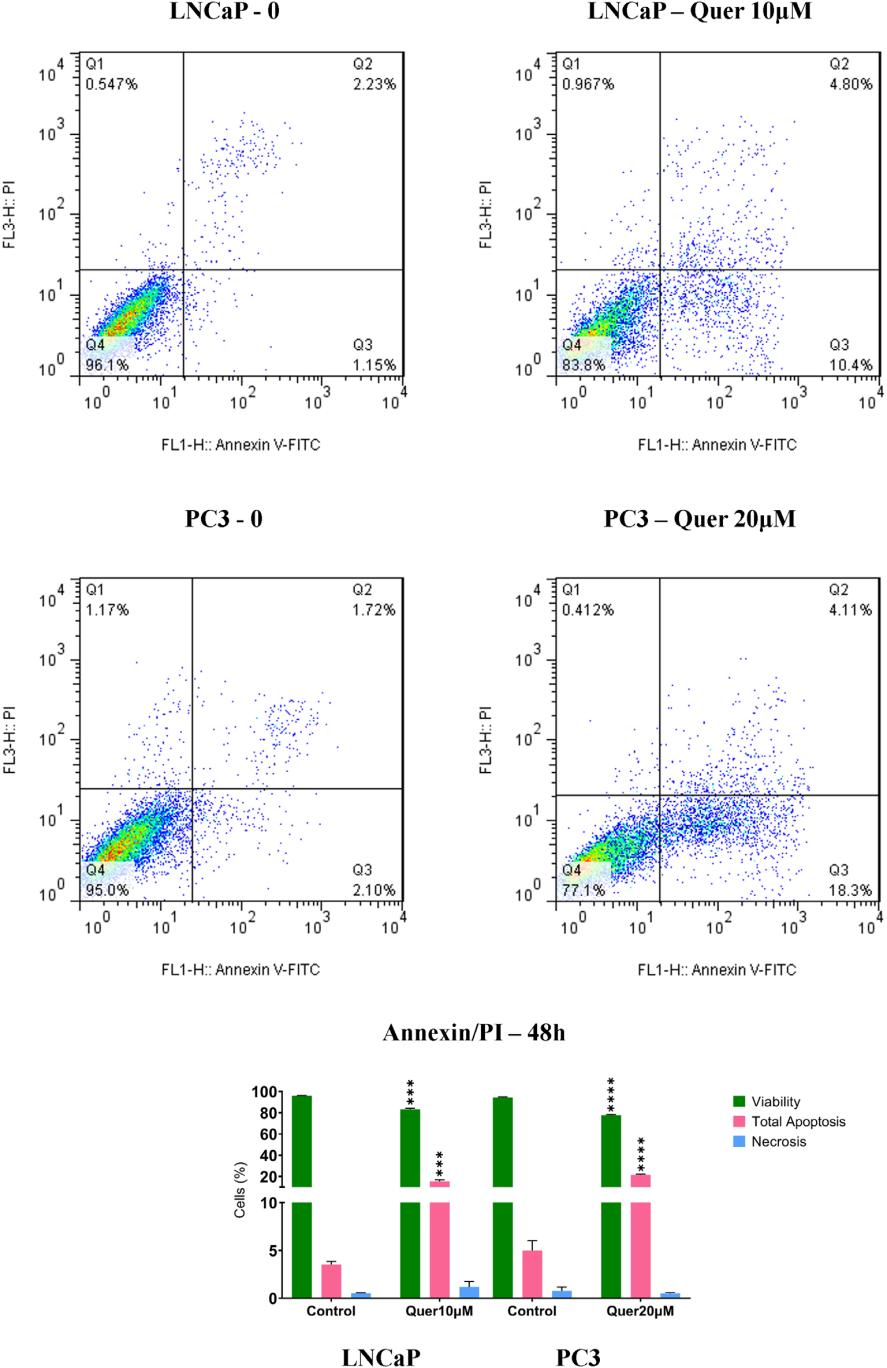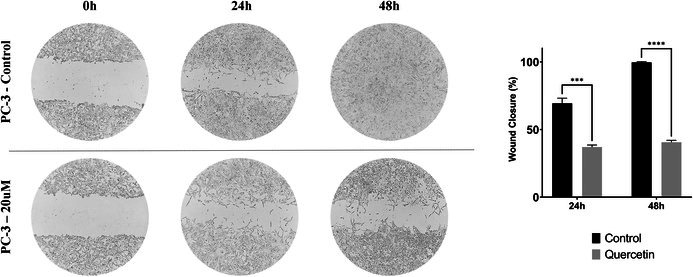# Correction to ‘Quercetin Can Be a More Reliable Treatment for Metastatic Prostate Cancer Than the Localized Disease: An In Vitro Study’

**DOI:** 10.1111/jcmm.71009

**Published:** 2026-03-04

A. Mirzaei, R. Deyhimfar, H. Azodian Ghajar, R. Mashhadi, M. Noori, H. Dialameh, Z. Aghsaeifard, and S. M. Aghamir, ‘Quercetin Can Be a More Reliable Treatment for Metastatic Prostate Cancer Than the Localized Disease: An In Vitro Study,’ *Journal of Cellular and Molecular Medicine* 27, no. 12 (2023): 1725–1734.

In Figure 4 of the original article, the flow cytometry plots depicting untreated and quercetin‐treated prostate cancer cells were presented incorrectly. These errors arose from a technical issue during the manuscript preparation. The duplicated images were inadvertently originated from another study previously conducted by one of the authors. We sincerely apologise for this oversight. In addition, due to poor image quality, the scratch images in Figure 7 were replaced with high‐quality versions. The overall results, conclusions and figure captions of the article remain unchanged.